# Knowledge, attitude, behavior and practices on H1N1 among the heterogenous population of Tamil Nadu, India

**DOI:** 10.1186/1753-6561-5-S6-P82

**Published:** 2011-06-29

**Authors:** CR Parathasarathy, P Chitra Rajalaksmi, T Jeyaseelan Senthinath, P Revathi, A Uma, M Ismail, A Ahamathunisha, P Thirumalaikolundusubramanian

**Affiliations:** 1 Microbiology, Chennai Medical College Hospital & Research Centre,Trichy, India

## Introduction / objectives

Audio visual media (AVM) have disseminated abundant information on Swine flu (H1N1) during the 2009 pandemic. Hence it was decided to find out the Knowledge, Attitude, Behavior and Practices regarding Swine flu and to elicit the influence of AVM on the general public regarding preventive measures.

## Methods

Population sampling was carried out taking into account that sizeable numbers represented different population groups. An anonymous pre tested questionnaire (closed 16, open 1) was given to 2193 participants. Voluntary participation was encouraged. Data were analyzed using SPSS17.0 software.

## Results

Response rate was 98%. The Urban to Rural Population ratio in the survey was 1:1.2. Television (86%) was the main source of information. Knowledge on pandemic nature, symptoms, personal protective measures, treatment and preventive strategies were acceptable among 90.2, 96, 36, 12 & 31% respectively. None of them used face mask or hand wash, though known to 78 & 20% respectively. Literacy level was directly proportional to knowledge gained and retained. However, there was no statistical difference among gender or domicile status.

## Conclusion

AVM have sensitized the public on symptomatology and enhanced health seeking behavior but not stressed effectively on preventive measures. AVM disseminated information during an outbreak, but lacked reinforcement during quiescent period. So it is suggested that health authorities should constantly provide information choosing the right media to the right population group towards preparing them for similar outbreaks.

## Disclosure of interest

None declared.

